# Supplementary figures and images for: Remnant tissue enhances early postoperative biomechanical strength and infiltration of Scleraxis-positive cells within the grafted tendon in a rat anterior cruciate ligament reconstruction model
Source: PLoS One. 2023 Nov 8;18(11):e0293944. doi: 10.1371/journal.pone.0293944 (PMC10631660; doi:10.1371/journal.pone.0293944)

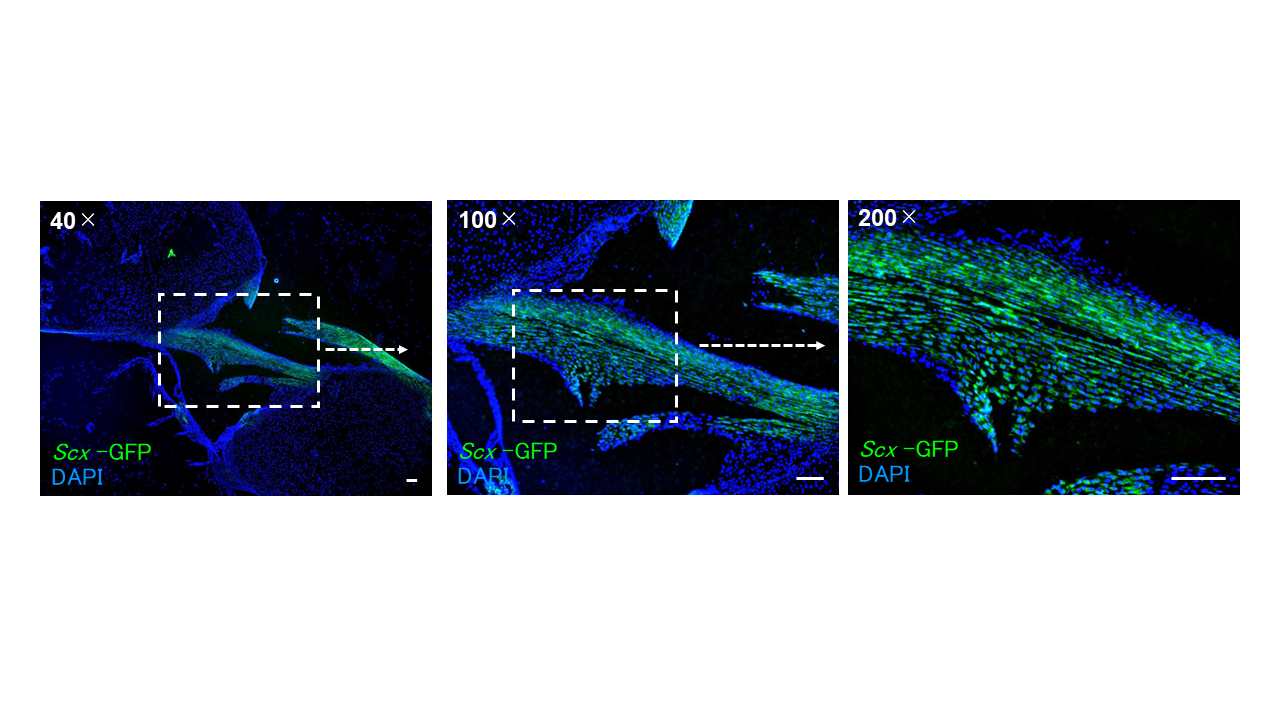

Supplement: S1 Fig — Knee joint tissues were collected from 1-day-old Scx-GFP Tg rats and stained with anti-GFP antibody, as a means to track Scx-expressing cells based on GFP fluorescence. Nuclei are DAPI-stained. Magnifications are as indicated. Bar, 100μm. (TIF) [file pone.0293944.s001.tif]

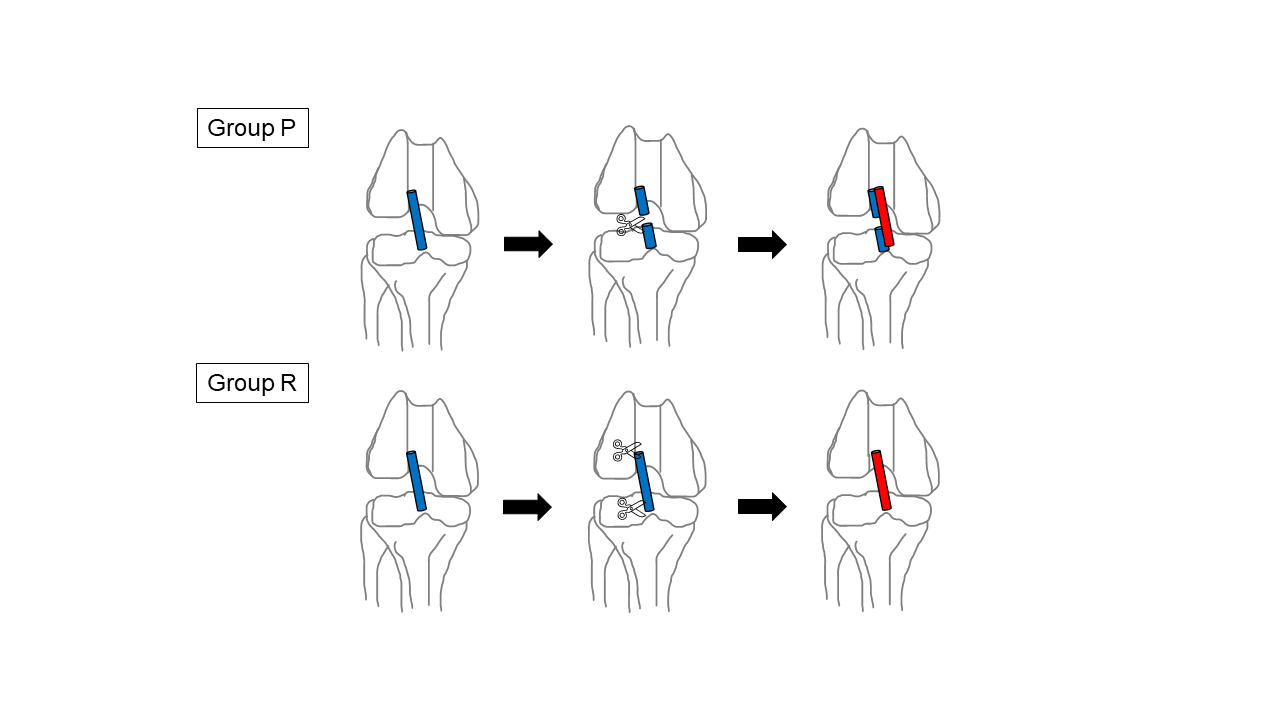

Supplement: S2 Fig — In remnant preservation models (Group P), the ACL was dissected at the center of the parenchyma, bone tunnels were created in the vicinity of the dissected ACL, and the graft tendon was inserted. In remnant resection models (Group R), the ACL was dissected from bony attachments, and the graft tendon was inserted at the anatomic location of the ACL. In addition, for ACL tear models, the ACL was dissected at the center of the parenchyma, and closed wound models were used. (TIF) [file pone.0293944.s002.tif]

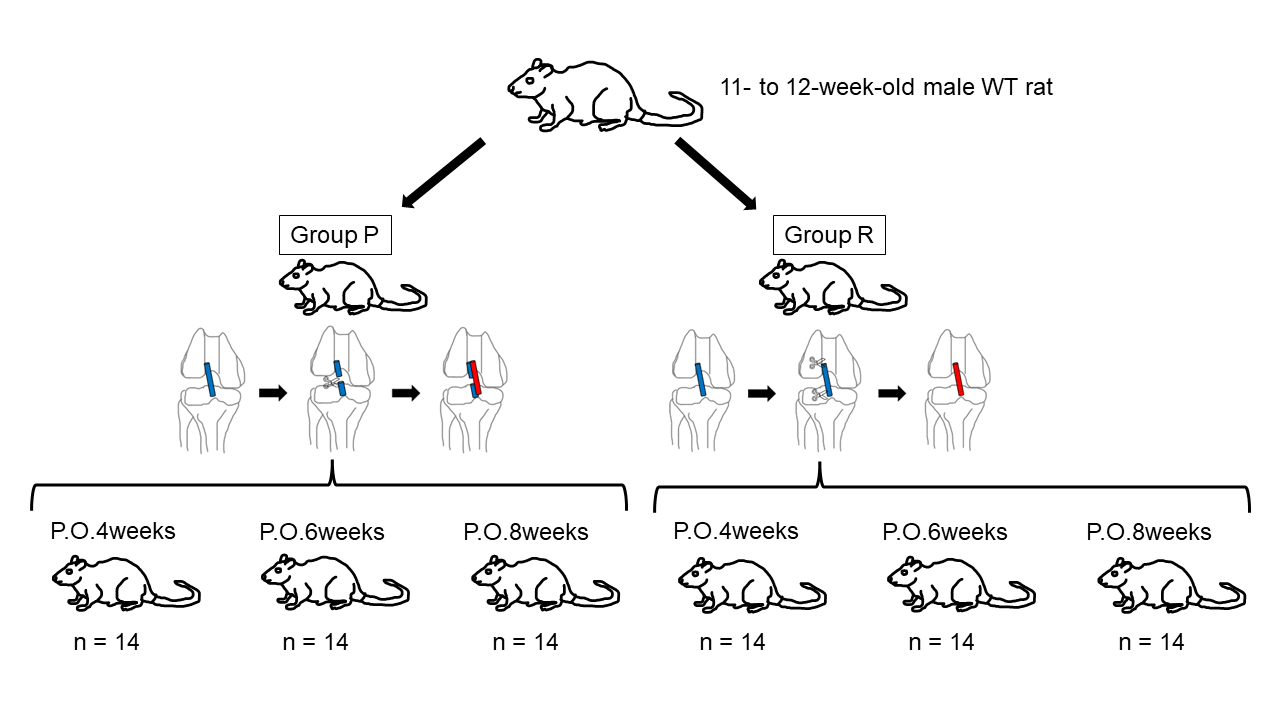

Supplement: S3 Fig — ACL reconstruction models were divided into remnant preservation models and resection models. For both, knee joint tissues from wild-type male rats were collected at 4, 6, or 8 weeks postoperatively and subjected to tensile tear testing. As indicated, sample size in all groups was 14 rats per group. (TIF) [file pone.0293944.s003.tif]

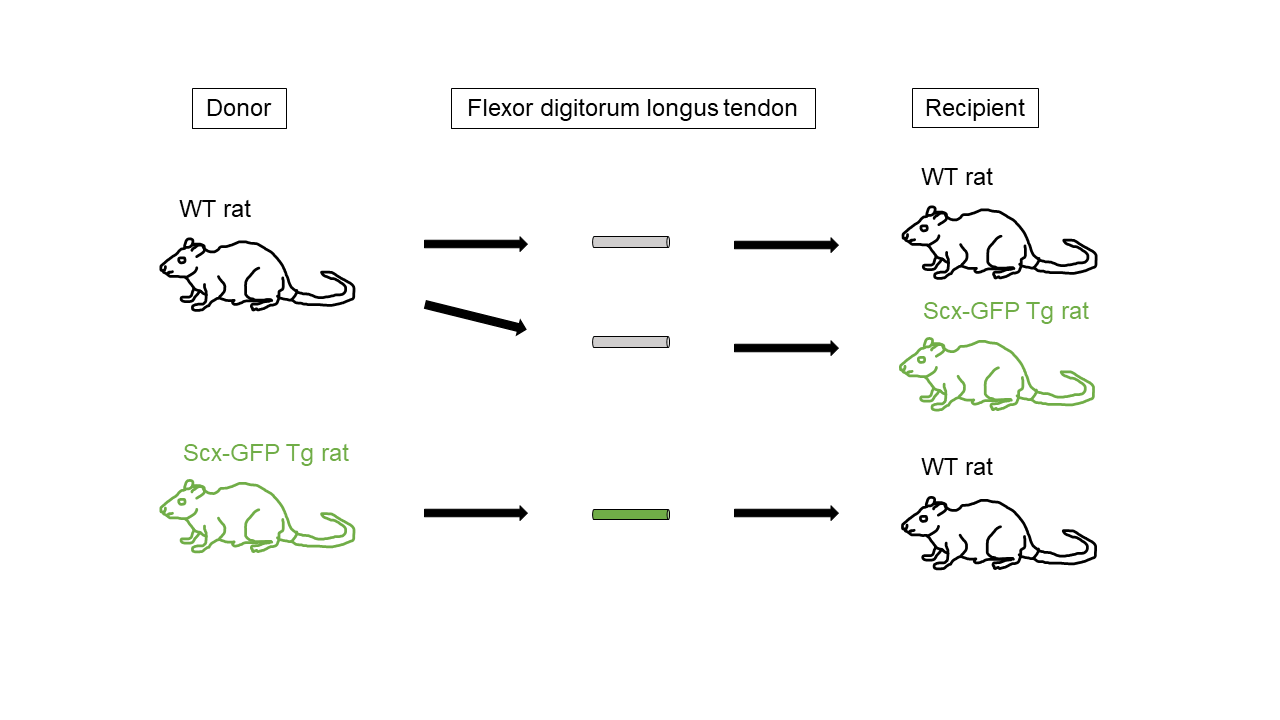

Supplement: S4 Fig — To determine the origin of Scx-expressing cells, three types of ACL allogeneic reconstruction models were created using 11 to 12-week-old Scx-GFP Tg or wild-type male rats. In the first, wild-type rats are transplanted with grafts from wild-type rats. In the second, Scx-GFP Tg rats are transplanted with grafts from wild-type rats, and in the third, wild-type rats are transplanted with grafts from Scx-GFP Tg rats. (TIF) [file pone.0293944.s004.tif]

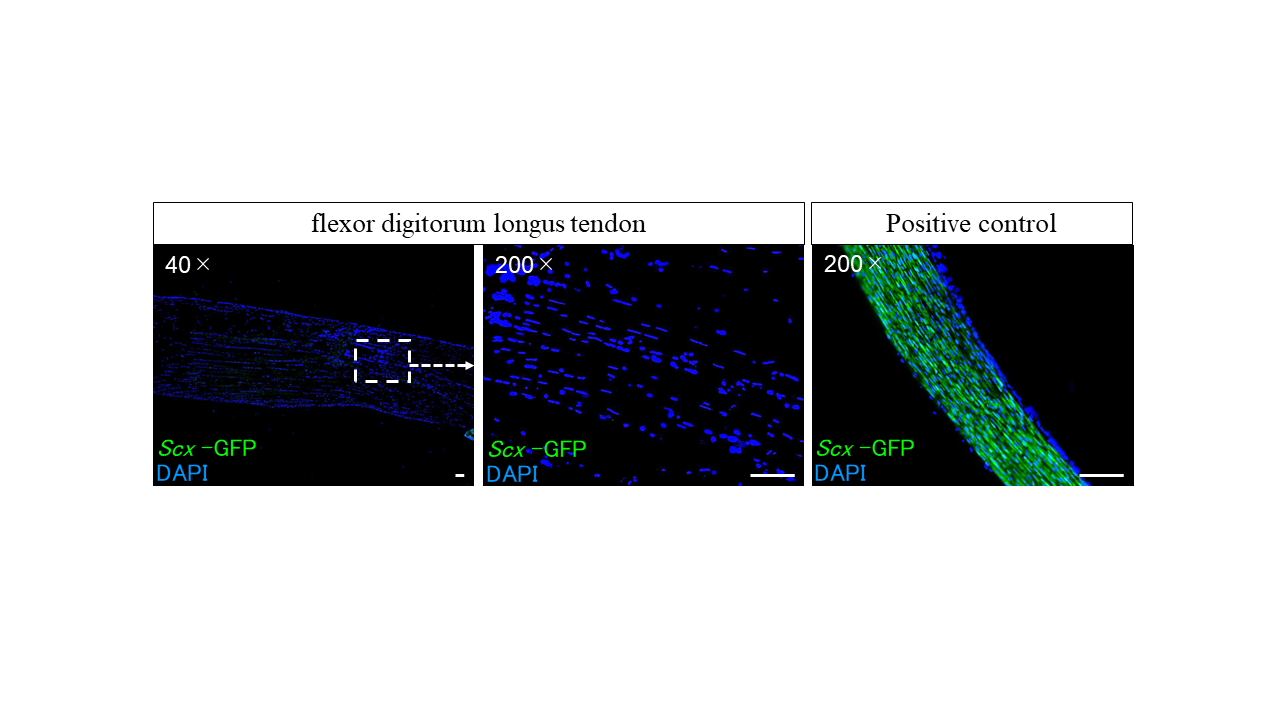

Supplement: S5 Fig — Long digitorum flexor tendons from 11 to 12-week-old Scx-GFP Tg rats were harvested, paraformaldehyde-fixed, freeze-embedded, and sectioned (5μm). Sections were then stained with anti-GFP antibody to detect Scx-expressing cells, as reflected by GFP positivity, within the grafted tendon prior to implantation. Nuclei are DAPI-stained. Note that no GFP-positive cells are present in the flexor digitorum longus tendon prior to transplant. As a positive control, we used Achilles tendons from 1-day-old Scx-GFP Tg rats. Magnifications are as indicated. Bar, 100μm. (TIF) [file pone.0293944.s005.tif]
