# Supplementary material for: Remnant tissue enhances early postoperative biomechanical strength and infiltration of Scleraxis-positive cells within the grafted tendon in a rat anterior cruciate ligament reconstruction model
Source: PLoS One. 2023 Nov 8;18(11):e0293944. doi: 10.1371/journal.pone.0293944 (PMC10631660; doi:10.1371/journal.pone.0293944)
Supplement: S1 File — (DOCX) [file pone.0293944.s006.docx]

Figure 1.

Figure 2.

Figure 3B.

Figure 3C.
